# Two Weeks of High-Intensity Interval Training in Combination With a Non-thermal Diffuse Ultrasound Device Improves Lipid Profile and Reduces Body Fat Percentage in Overweight Women

**DOI:** 10.3389/fphys.2019.01307

**Published:** 2019-10-22

**Authors:** Christophe Hausswirth, Laurie-Anne Marquet, Xavier Nesi, Katie Slattery

**Affiliations:** ^1^Laboratoire Motricité Humaine Expertise Sport Santé, University of Côte d’Azur, Nice, France; ^2^BeScored Institute, Sophia Antipolis, France; ^3^Human Performance Research Centre, University of Technology Sydney, Ultimo, NSW, Australia; ^4^TechnoSport, Aix-Marseille University, Marseille, France

**Keywords:** exercise, weight loss, HIIT, low-frequency, low-intensity ultrasound, fitness

## Abstract

This study evaluated the effectiveness of an innovative strategy which combined low-frequency ultra sound (LOFU) with high-intensity interval training (HIIT) to improve physical fitness and promote body fat loss in overweight sedentary women. A placebo controlled, parallel group randomized experimental design was used to investigate the efficacy of a 2-week combined LOFU and HIIT program (3 sessions per week). Participants were allocated into either the Experimental HIIT group (HIIT_EXP_, *n* = 10) or Placebo HIIT group (HIIT_PLA_, *n* = 10). Baseline exercise testing (maximal oxygen uptake, lower limb strength and substrate oxidation test), dietary assessment, anthropometric measures and blood sampling were completed in week 1 and repeated in week 4 to determine changes following the program (Post-HIIT). During each training session, the HIIT_EXP_ and HIIT_PLA_ groups wore a non-thermal diffuse ultrasound belt. However, the belt was only switched on for the HIIT_EXP_ group. Delta change scores were calculated for body weight, body fat percentage (Fat%), muscle mass, $\dot{\text{V}}$O_2__max_, hip and waist circumferences, and all lipid variables from Baseline to Post-HIIT. Statistical analysis was completed using a repeated-measures factorial analysis of variance by group (HIIT_PLA_ and HIIT_EXP_) and time (Baseline and Post-HIIT). Results showed significant improvements in maximal oxygen uptake (HIIT_EXP_; Baseline 24.7 ± 5.4 mL kg^–1^ min^–1^, Post-HIIT 28.1 ± 5.5 mL kg^–1^ min^–1^ and HIIT_PLA_; Baseline 28.4 ± 5.9 mL kg^–1^ min^–1^, Post-HIIT 31.4 ± 5.5 mL kg^–1^ min^–1^) for both groups. Significant decreases in Fat% (HIIT_EXP_; Baseline 32.7 ± 3.2%, Post-HIIT 28.9 ± 3.5% and HIIT_PLA_; Baseline 28.9 ± 3.5%, Post-HIIT 28.9 ± 3.4% kg), waist circumference (HIIT_EXP_; Baseline 95.8 ± 9.6 cm, Post-HIIT 89.3 ± 8.9 cm and HIIT_PLA_; Baseline 104.3 ± 3.5 cm, Post-HIIT 103.6 ± 3.4 cm) and triglycerides (HIIT_EXP_; −29.2%, HIIT_PLA_; −6.7%) were observed in the HIIT_EXP_ group only. These results show that HIIT combined with LOFU was an effective intervention to improve body composition, lipid profile, and fitness. This combined strategy allowed overweight, sedentary women to achieve positive health outcomes in as little as 2 weeks.

## Introduction

Sedentary behavior and physical inactivity are closely associated with the development of risk factors for metabolic syndrome including glucose intolerance, insulin resistance, hypertension, dyslipidemia, and obesity (Eckel et al., 2010). Fat localization, particularly abdominal adipose tissue, is also a major determinant in the occurrence of metabolic disorders (Wajchenberg, 2000). Due to the increasing prevalence of overweightness and obesity, combined with the significant health costs and economic burden of sedentary behavior, it is important to investigate strategies that induce a loss of body fat and promote long-term weight management (Ogden et al., 2006). In this context, a balanced diet and physical activity interventions are the main approaches used to reduce body fat and improve an individual’s blood lipid profile (Donnelly et al., 2009; Johns et al., 2014). Indeed, comparisons between sedentary and physically active groups in cross-sectional studies have shown the positive influence of exercise on blood lipid profile (Durstine et al., 2001). Moderate-intensity continuous training (MICT) is currently recommended to promote weight loss (Donnelly et al., 2009), as prolonged exercise has been demonstrated to increase fat mobilization and oxidation (Katzmarzyk et al., 2001; Lazzer et al., 2017). However, evidence suggests that high-intensity interval training (HIIT) is also an effective strategy for reducing body fat (Boutcher, 2011; Maillard et al., 2018) and could lead to a greater loss in fat mass than MICT (Wewege et al., 2017).

High-intensity interval training sessions involve short periods of high-intensity exercise [80–100% of maximum heart rate (HRmax)] interspersed with passive rest, or low intensity exercise for recovery (Weston et al., 2014). HIIT programs have shown similar fitness benefits to MICT, but can be completed in a shorter amount of time and have been perceived as more enjoyable, which can promote exercise adherence (Bartlett et al., 2011). Moreover, several studies have shown that HIIT programs have a positive effect on fat loss (Tremblay et al., 1994; Mourier et al., 1997; Gibala and McGee, 2008; Tjonna et al., 2009; Boutcher, 2011) due to metabolic adaptations such as increased fat oxidation (Talanian et al., 2007), increased oxidative metabolism (Tremblay et al., 1994; Gibala and McGee, 2008) and enhanced insulin sensitivity (Babraj et al., 2009). For example, Tremblay et al. (1994) compared the effect of a 20-week MICT and HIIT program (5 sessions per week) on body fat loss. A nine-fold greater reduction in subcutaneous body fat was observed following the HIIT program compared to MICT, when the exercise sessions were corrected for total energy cost. Moreover, a significantly greater expression of the enzyme 3-hydroxyacyl-coenyme A dehydrogenase which is involved in the β-oxidation pathway was observed following HIIT. This lends support to the ability of HIIT to improve fat oxidation to a greater extent than MICT. Similar findings were reported by Trapp et al. (2008) using a shorter training duration (20-min per session vs. 40-min) where a 15-week (3 sessions per week) led to a significant decrease in total fat mass (−2.50 ± 0.83 kg) and reduction in central abdominal fat (−0.15 ± 0.07 kg) compared to the MICT and sedentary control groups. Collectively, these results demonstrate the efficacy of HIIT to improve fitness and reduce body fat. However, there are other non-exercise based inventions that may also promote fat loss and further improve blood lipid profile.

Low-intensity (up to 17.5 W/cm^2^), low-frequency (20–200 kHz) ultrasound (LOFU) therapy is a safe, non-invasive body-contouring technique to reduce fat mass (Hotta, 2010; Tonucci et al., 2014; Friedmann, 2015; Juhasz et al., 2018). This technology uses ultrasound to produce a mechanical stress that disrupts the cellular membrane of adipose tissue (Hotta, 2010). Specifically, microcavities are created that subsequently cause cell destruction and fat liquefaction (Tonucci et al., 2014). These mobilized fat cells are metabolized in the liver or removed via catabolic processes. While LOFU is an emerging tool that requires further well-controlled studies, the initial findings are promising. Studies using LOFU have reported a significant reduction in abdominal circumference (−2.1 cm) (Tonucci et al., 2014), decreased total fat mass (−3.5%) and reduced subcutaneous adipose tissue (mean −2.4%) (Milanese et al., 2014) across a series of treatments.

The aim of the present study was to evaluate an innovative weight loss strategy coupling LOFU with a HIIT program on fitness level, body composition and lipid profile in sedentary, overweight women. We hypothesized that in addition to the benefits of HIIT on fat metabolism and fitness, the increased fat mobilization induced by LOFU would lead to an increased uptake of fat by the exercising muscles and further reductions in fat mass and body weight. It was also anticipated that the added LOFU would lead to comparable changes in body composition and lipid profile following a short-term 2-week training intervention as a longer 15 to 20-week training intervention.

## Materials and Methods

### Participants

Twenty-three healthy, sedentary women aged between 20 and 49 years old were initially recruited. Participants volunteered after being fully informed of the study purpose, protocols, procedures, and potential risks of involvement. All participants provided informed consent and the investigation was approved by a local ethic committee (University of Nice) in compliance with the Declaration of Helsinki. On the initial visit to the laboratory, each subject underwent a physical examination conducted by a cardiologist. Based on this examination, participants with high blood pressure, extrasystoles or serious heart rhythm abnormalities were excluded from the investigation. Participants were then classified as sedentary with self-reported ≤ 15-min of moderate aerobic exercise per week (Bartfay and Bartfay, 2014). A further medical screening was performed and participants with recent infections, muscular and/or joint disability, smokers and those using hormonal replacement therapy, antioxidant supplementation or taking medication affecting lipid or lipoprotein metabolism were excluded from the study. The phase of the menstrual cycle was also reported and participants began the baseline exercise testing while in the follicular phase. Participants were then randomly allocated via random number allocation into one of two groups. Due to illness, three participants withdrew from the investigation (Experimental *n* = 2, Placebo *n* = 1). The, we analyzed 20 participants: 10 for the Experimental HIIT group (HIIT_EXP_) and 10 for the Placebo HIIT group (HIIT_PLA_). There was no significant difference in body composition, anthropometric or fitness measures between the groups before the study commenced.

### Procedures

**FIGURE 1:**
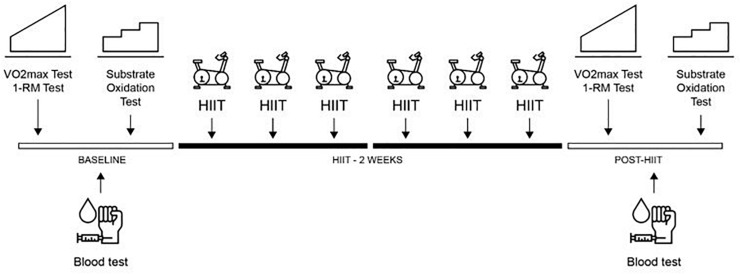
Overview of the experimental protocol. One repetition maximum on leg press (1-RM Test), high-intensity interval training (HIIT), Maximal oxygen test ($\dot{\text{V}}$O_2max_ Test).

A placebo controlled, parallel group randomized experimental design was used to investigate the efficacy of a combined HIIT and LOFU program. The investigation was conducted over a period of 4 weeks. Baseline exercise testing (maximal oxygen uptake, lower limb strength, and substrate oxidation test), dietary assessment, body composition, anthropometric measures and blood sampling were completed in week 1 and repeated in week 4 to determine changes following the program (Post-HIIT). Each group (HIIT_EXP_ and HIIT_PLA_) were asked to complete the same HIIT program in weeks 2 and 3 (3 sessions per week). During each training session, the HIIT_EXP_ and HIIT_PLA_ groups wore a non-thermal diffuse ultrasound belt (Slim Sonic L-1440, Lausanne, Switzerland) using the technology of Sonic Resonance^®^. This device has been described in a previous study (Hafiz et al., 2014). The system operated at a frequency ranging from 30 to 42 kHz. The intensity applied was fixed between 5 to 8 W/cm^2^ cavitation power. The device weighed 1.6 kg and was placed on each participant’s waist. It was switched on for the HIIT_EXP_ group and switched off for the HIIT_PLA_ group for 45-min during exercise. Participants were asked to maintain their typical dietary practices throughout the investigation and were not informed that energy intake was a variable that would be assessed. An overview of the experimental design is outlined in Figure 1.

### Exercise Testing Sessions

#### Evaluation of the Lower Limb Maximal Strength

Measurements were undertaken 2-day before and 2-day after the six HIIT sessions. Participants were familiarized with the testing procedure during a standardized warm-up which consisted of jump squats (2 sets of 5 reps), countermovement jumps (2 sets of 5 reps) and submaximal isokinetic bilateral lower limb extensions at 40% 1RM (2 sets of 15 reps) on a digital leg press (eGym, Munich, Germany). Muscle strength was measured as peak force (F_max_) during an isokinetic maximal voluntary bilateral lower limb extension on a digital leg press during a single concentric movement. Velocity was regulated to move through the full range of motion in 2-s. Customized software was then used to translate the torque values into kilograms and calculate the F_max_, irrespective of joint angle. Strict care was taken to ensure identical test protocols for all participants, which included standardized verbal encouragement and visual feedback provided by a real-time display of the force output. Successive trials were performed until F_max_ could not be improved any further, which typically included seven to nine attempts (Aagaard et al., 2000).

#### Maximal Oxygen Uptake Test

One week before and 2 to 4 days after the training period, participants undertook an incremental cycle test to exhaustion on a stationary electromagnetically braked cycle ergometer (Monark LC6 novo, Vansbro, Sweden) to determine maximal oxygen uptake ($\dot{\text{V}}$O_2_max) and maximal aerobic power (MAP). To minimize the effects of diet on physical performance, participants standardized their diet in the 24-h prior to each maximal oxygen uptake test. Furthermore, 2-h prior to each maximal oxygen uptake test, participants consumed a meal which contained at least 2 g/kg/body mass of carbohydrate. This meal was recommended by a dietician based on the participant’s 3-day dietary analysis. Following a 6-min warm-up at 60 W, the workload was increased at increments of 20 W every 2-min until exhaustion. During this test, oxygen uptake ($\dot{\text{V}}$O_2_) and expiratory flow ($\dot{\text{V}}$E) were collected and respiratory exchange ratio (RER) was calculated from the ratio between oxygen uptake and carbon dioxide output ($\dot{\text{V}}$CO_2_⋅$\dot{\text{V}}$O_2_^–1^) using a breath by breath gas analyzer (Cosmed Quark CPET, Rome, Italy). Heart rate (HR) was recorded using a chest belt (Cosmed wireless HR monitor, Rome, Italy). The criteria used for the determination of $\dot{\text{V}}$O_2_max were threefold: a plateau in $\dot{\text{V}}$O_2_ despite an increase in power output, a RER above 1.1, and a HR above 90% of the predicted maximal HR (Howley et al., 1995). Expired gases and HR values were averaged every 10-s. $\dot{\text{V}}$O_2__max_ and MAP were defined as the average of the highest consecutive $\dot{\text{V}}$O_2_ and power output values recorded during a 1-min period.

#### Substrate Oxidation Assessment

Following the maximal oxygen uptake test, participants returned to the laboratory a minimum of 24–48-h later at the same time of day after an overnight (12-h) fast to determine substrate oxidation. Before this trial, they were required to record all food and drink ingested in the previous 24-h on a dietary log and asked to confirm that they were indeed in a fasted state. This form was photocopied and returned to each subject, and they were required to replicate this dietary intake before the Post-HIIT assessment of substrate oxidation. Before exercise, resting gas exchange data were acquired for 4-min to ensure that participants were not hyperventilating. Exercise consisted of 4-min of cycling (Monark LC6 novo, Vansbro, Sweden) at 40 W followed by 20 W increases in intensity every 3-min until RER remained higher than 1.0 for at least 60-s. This protocol is similar to the one employed by Achten et al. (2002) but adapted for sedentary women. Cadence was maintained between 60 and 80 rpm. Gas exchange data (Cosmed Quark CPET, Rome, Italy) and HR (Cosmed wireless HR monitor, Rome, Italy) were continuously obtained, and the last 2-min of gas exchange data from each stage was averaged to calculate $\dot{\text{V}}$O_2_ and $\dot{\text{V}}$CO_2_ and to determine RER. Specifically, whole body rates of carbohydrate (CHO) and fat oxidation (g min^–1^) were calculated from $\dot{\text{V}}$O_2_ and $\dot{\text{V}}$CO_2_ values measured during the submaximal cycling test using the non-protein RER values and according to standard equations (Jeukendrup and Wallis, 2005): CHO oxidation = 4.210 ($\dot{\text{V}}$CO_2_) – 2.962 ($\dot{\text{V}}$O_2_) and fat oxidation = 1.695 ($\dot{\text{V}}$O_2_) – 1.701 ($\dot{\text{V}}$CO_2_).

### Dietary Intake Assessment

To minimize a possible nutritional bias, all participants were instructed to maintain their accustomed dietary habits throughout the investigation from 3-day prior to Baseline testing in Week 1 to the completion of the post-testing in Week 4. No attempt was made to modify the nutrient composition of the individual’s diets or their total energy intake. However, the participants all worked for the same organization and were asked to eat breakfast, lunch and snacks from the cafeteria each day of the intervention. Participants were instructed to record their dietary intake for 3-day before the Baseline maximal oxygen uptake test, including 1 weekend day, and for 3-day during the second week of training (HIIT_*WK*__2_). These 3-day dietary records were analyzed for total energy intake and for composition of carbohydrates, fats and protein using a commercially available computer software program (Nutrilog, Marans, France).

### Body Composition and Anthropometric Assessments

Body composition (body weight, lean mass, and body fat percentage [Fat%]) was assessed following a 12-h fast with a bioelectrical impedance analysis device (Tanita model MC780 MA; Tanita Europe B.V., Amsterdam, Netherlands) that has been previously validated compared to dual-energy x-ray absorptiometry [fat mass (kg) ICC: 0.88; Lin C: 0.89] (Verney et al., 2016), in compliance with the manufacturer’s guidelines. Anthropometric assessment included height, hip and waist circumference. Circumference measurements were evaluated at the same height and under constant tension by the same tester using a calibrated tape measure and standardized technique (Bernritter et al., 2011). Participants wore light shorts and stood with arms crossed and hands tucked under the axillae and were instructed to relax their abdominal muscles, exhale, and hold their exhalation throughout each measurement. Measures were taken in duplicate and the mean value was recorded. These assessments were performed at Baseline and Post-HIIT.

### Blood Analyses

Blood samples were collected before and after 2 weeks of HIIT, following a 12-h fasting period. Participants were instructed to avoid alcohol and strenuous physical activity 48-h before collection. Samples were collected from the antecubital region into 4 ml ethylenediaminetetraacetic acid (EDTA) anticoagulant and serum separator tubes (SSTs). Then plasma samples were immediately transferred to pre-chilled microtubes and SST were immediately placed in ice and centrifuged at 3000 rpm for 10-min. All samples were stored at −20°C for later analyses at a commercial laboratory within 2 weeks of the completion of the training program. Baseline and Post-HIIT samples were analyzed in a single laboratory session to reduce interassay variation. Glycerol was analyzed by fluorometric techniques (Randox Laboratories, County Antril, United Kingdom) (Foster et al., 1978), triglycerides (TGs) (Bucolo and David, 1973) and non-esterified fatty acids (NEFAs) were analyzed by an enzymatic colorimetric technique (NEFA kit, Biomnis, Paris, France) by combining acetyl coenzyme A synthetase and acetyl coenzyme A oxidase. The fasting glucose concentration was analyzed using the Atellica^®^ CH Glucose Oxidase test (Siemens Healthcare SA, Renens, Switzerland) (Fossati et al., 1983).

### High-Intensity Interval Training Protocol (HIIT)

HIIT was performed on Monark cycle ergometers (Monark LC6 novo, Vansbro, Sweden), three times per week, for 2 weeks. Participants completed all training sessions at a similar time of day. Two familiarization sessions were completed prior to the Baseline $\dot{\text{V}}$O_2_max test to allow the participants to become accustomed to wearing the LOFU device while exercising for 30-min at a low intensity (RPE 11-12) (Borg, 1982). Training intensity was controlled through the maximal HR (HRmax) obtained during the maximal oxygen uptake test. The HIIT protocol was adapted from the training program previously described by Steckling et al. (2016). All sessions began with a 10-min warm-up at 60% HRmax, included a cool-down at 60% HRmax and total training session time was 45-min. Sessions 1 and 2 consisted of eight, 2-min intervals at 90% HRmax with 2-min active recovery at 60% HRmax between each interval. Sessions 3 and 4 consisted of six, 3-min intervals at 90% HRmax with 2-min active recovery at 60% HRmax between each interval. Sessions 5 and 6 consisted of five, 4-min intervals at 90% HRmax with 2-min active recovery at 60% HRmax between each interval. All sessions were supervised by staff.

### Participant Satisfaction

At the end of the training period, a self-administrated questionnaire on customer satisfaction was completed [Client Satisfaction Questionnaire (CSQ-8)] (Attkisson and Zwick, 1982). The CSQ-8 can be easily scored and consists of eight items designed to measure client satisfaction with different services. Each item of the CSQ-8 can be scored from 1 to 4. The final score is calculated by adding up the individual items’ scores (minimum satisfaction = 8 and maximum satisfaction = 32).

### Statistical Analysis

All data were stored in an electronic database and analyzed using specialized statistical software (SPSS v20.0, Chicago, IL, United States). Results are expressed as mean ± standard deviation (SD). Delta change scores were calculated for body weight, Fat%, muscle mass, $\dot{\text{V}}$O_2_max, hip and waist circumferences, and all lipid variables from Baseline to Post-HIIT. The normality of distribution for each variable was tested using the Shapiro–Wilk test. Statistical analysis was completed using a repeated-measures factorial analysis of variance (ANOVA) by group (HIIT_PLA_ and HIIT_EXP_) and time (Baseline and Post-HIIT). If significant main effects were observed, a Tukey’s Honest Significant Difference test was performed as *post hoc* analysis to further discern differences. When assumptions of normality or homogeneity of variances were not met, the data was log-transformed before analysis. Means were then de-transformed back to their original units. The criteria to interpret the magnitude of effect size was > 0.2 small, > 0.5 moderate, > 0.8 large, and > 1.3 very large (Cohen, 1988; Rosenthal, 1996). An *a priori* sample analysis revealed 10 pairs of subjects was the minimum required in a matched pair design to be able to reject the null hypothesis that this response difference is zero with probability (power) 0.8. The Type I error probability associated with this test of this null hypothesis is 0.05 (G^∗^Power version 3.1.3, Universität Kiel, Germany). Statistical significance was accepted at *P* < 0.05. Correlation analysis was employed to explore relationships among these change scores.

## Results

### Exercise Testing Sessions

#### Evaluation of the Lower Limb Maximal Force (F_max_)

Both groups improved their F_max_ (HIIT_EXP_: 16.0%; HIIT_PLA_: 14.4%) with no significant difference between groups (*P* = 0.77) (Table 1).

**TABLE 1:** Mean ± SD values for fitness measures at Baseline and following the training intervention (Post-HIIT) in Placebo (HIIT_PLA_) and Experimental (HIIT_EXP_) groups.

**Parameters**		**Baseline**	**Post-HIIT**	**Change from baseline to Post-HIIT (%)**	**Cohen’s *d***
Maximal oxygen uptake (mL kg^–1^ min^–1^)	HIIT_PLA_	28.4 ± 5.9	31.4 ± 5.5^‡^	10.4	0.51
	HIIT_EXP_	24.7 ± 5.4	28.1 ± 5.5^‡^	13.6	0.61
Maximal aerobic power (W)	HIIT_PLA_	154.5 ± 30.5	170.0 ± 30.8^‡^	10.0	0.50
	HIIT_EXP_	145.0 ± 45.4	160.0 ± 41.8^‡^	10.3	0.34
Maximal heart rate (bpm)	HIIT_PLA_	185.4 ± 7.8	186.8 ± 8.2	0.7	0.17
	HIIT_EXP_	186.9 ± 9.5	188.1 ± 9.3	0.6	0.12
Lower limb maximal force (kg)	HIIT_PLA_	178.6 ± 37.0	207.3 ± 37.3^‡^	16.0	0.77
	HIIT_EXP_	179.9 ± 33.7	205.9 ± 37.3^‡^	14.4	0.73

*^‡^Different from Baseline (P < 0.01). Percentage change from Baseline to Post-HIIT. The criteria to interpret the magnitude of the effect size were as follows; >0.2 small, >0.5 moderate, >0.8 large, and >1.3 very large).*

#### Maximal Oxygen Uptake Test

Both groups showed similar improvements in $\dot{\text{V}}$O_2_max (HIIT_EXP_: 13.6%; HIIT_PLA_: 10.4%, *P* < 0.01) and their MAP (HIIT_EXP_: 10.3%; HIIT_PLA_: 10.0%, *P* < 0.01), following the HIIT training program (Table 1). However, no significant difference was observed between groups in $\dot{\text{V}}$O_2_max (*P* = 0.72), MAP (*P* = 0.95) or HRmax (*P* = 0.30).

#### Substrate Oxidation Assessment

**FIGURE 2:**
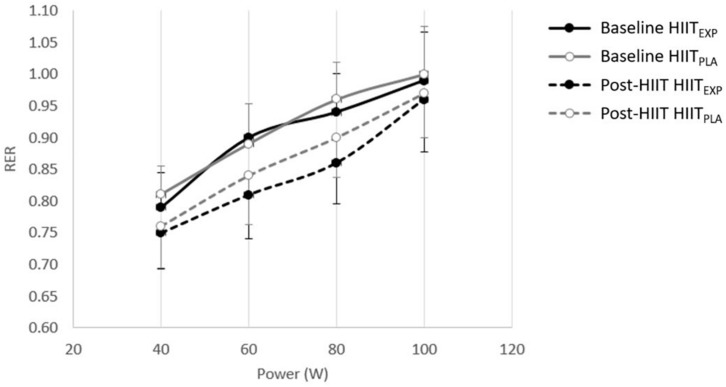
Change in RER (mean ± SD) before (Baseline) and after (Post-HIIT) the training program in Placebo (HIIT_PLA_) and Experimental (HIIT_EXP_) groups.

**FIGURE 3:**
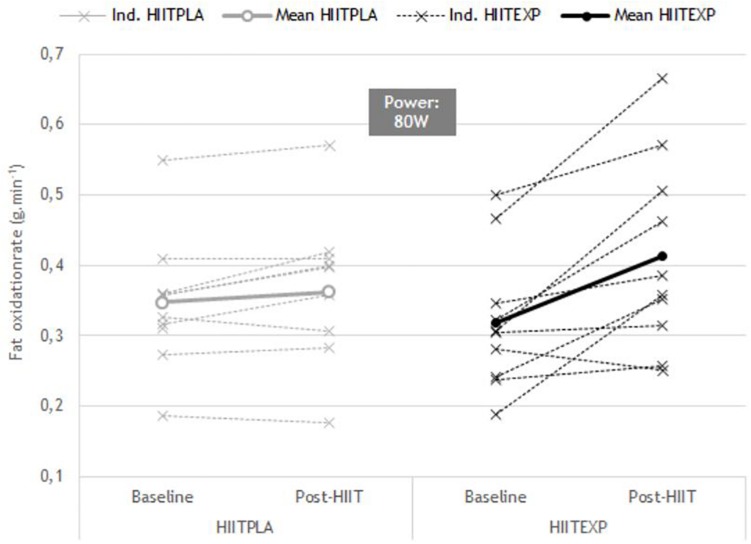
Individual change in RER (mean ± SD) before (Baseline) and after (Post-HIIT) the training program at 60 W in Placebo (HIIT_PLA_) and Experimental (HIIT_EXP_) groups.

**FIGURE 4:**
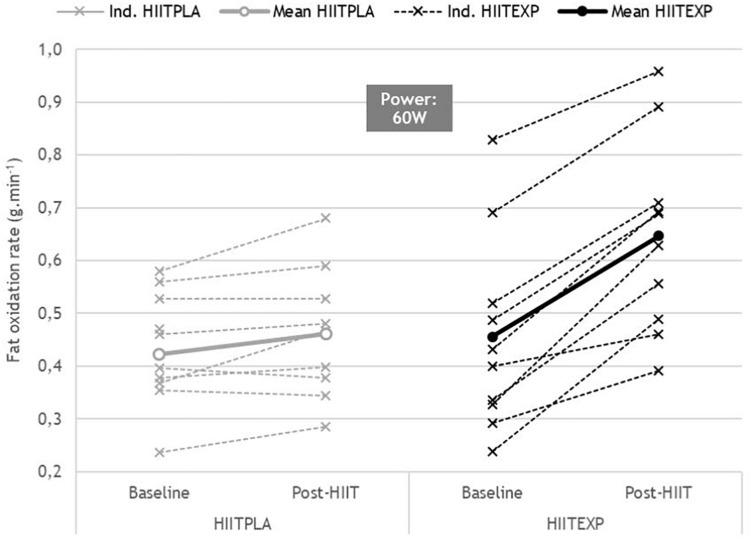
Individual change in RER (mean ± SD) before (Baseline) and after (Post-HIIT) the training program at 60 W in Placebo (HIIT_PLA_) and Experimental (HIIT_EXP_) groups.

Across all participants, the change in RER (Figure 2) in response to training was only examined up to 100 W in HIIT_PLA_ (*n* = 10) and in HIIT_EXP_ (*n* = 9), as many women did not attain a power output greater than 120 W during the Baseline maximal oxygen uptake test. No significant difference was observed between groups. However, there was a tendency toward a greater rate of fat oxidation in HIIT_EXP_ following combined HIIT and LOFU at 60 W (Baseline: 0.45 ± 0.03 g min^–1^ to Post-HIIT: 0.65 ± 0.4 g min^–1^) (Figure 3) and at 80 W (Baseline: 0.31 ± 0.02 g min^–1^ to Post-HIIT: 0.41 ± 0.4 g min^–1^) (Figure 4).

### Dietary Intake Assessment

Nutrient intake was similar (*P* > 0.05) for both groups at Baseline (Table 2). Results revealed no effect of HIIT (*P* = 0.29) or difference between groups (*P* = 0.37) for total calorie intake. However, a significant increase (16.6%, *P* < 0.05) in CHO intake was observed in HIIT_PLA,_ from 46.8 ± 5.9% at Baseline to 54.6 ± 4.4% at HIIT_*WK*__2_. In addition, a significant decrease (17.6%, *P* < 0.05) in fat intake was observed in HIIT_EXP,_ from 36.3 ± 6.4% at Baseline to 29.9 ± 3.2% at HIIT_*WK*__2_. No significant difference was observed for protein (*P* > 0.05) irrespective of group or period.

**TABLE 2:** Mean ± SD values for changes in dietary intake at Baseline and during week 2 of HIIT training program (HIIT_WK__2_) in Placebo (HIIT_PLA_) and Experimental (HIIT_EXP_) groups.

		**Baseline**	**HIIT_*WK*__2_**
Nutrient intake (kcal)	HIIT_PLA_	1589.4 ± 268.4	1655.5 ± 214.7
	HIIT_EXP_	1677.7 ± 246.8	1533.8 ± 212.5
Carbohydrate (%)	HIIT_PLA_	46.8 ± 5.9	54.6 ± 4.4^†^
	HIIT_EXP_	49.5 ± 7.1	51.9 ± 2.7
Fat (%)	HIIT_PLA_	36.4 ± 8.7	32.7 ± 7.6
	HIIT_EXP_	36.3 ± 6.4	29.9 ± 3.2^†^
Protein (%)	HIIT_PLA_	16.1 ± 4.6	12.7 ± 4.5
	HIIT_EXP_	14.1 ± 4.2	17.9 ± 3.7

*^†^Difference between Baseline and HIIT_*WK2*_ (P < 0.05).*

### Body Composition and Anthropometric Assessment

Changes in body composition and anthropometric measures are presented in Table 3. There was a significant interaction (group × time) effect for body weight (*P* < 0.01), with a significant −1.8% (*P* < 0.01) decrease in body weight for women in the HIIT_EXP_ group following the HIIT program. This decrease in body weight led to a decrease of BMI for the HIIT_EXP_ group (−1.9%; *P* < 0.01). No significant variation in body weight or BMI was observed in HIIT_PLA_ group (*P* > 0.05). Significant changes in body composition were also observed in the HIIT_EXP_ group only, with a −4.5% (*P* < 0.01) decrease in Fat%. In comparison, no change (0.2%, *P* > 0.05) in Fat% was observed in the HIIT_PLA_ group. No difference was noted in either group for lean mass (*P* > 0.05). An interaction effect (group × time, *P* < 0.01) was observed for the hip circumference with a significant decrease for HIIT_EXP_ group (hip: −4.1%, *P* < 0.01 vs. −0.7%, *P* > 0.05, for HIIT_EXP_ and HIIT_PLA__,_ respectively). Moreover, an interaction effect (group × time, *P* < 0.01) was observed also for waist circumference with a significant decrease for the HIIT_EXP_ group only (waist: −6.7%, *P* < 0.01 vs. −0.8%, *P* > 0.05, for HIIT_EXP_ and HIIT_PLA__,_ respectively).

**TABLE 3:** Mean ± SD values for body composition variables at Baseline and following the training intervention (Post-HIIT) in Placebo (HIIT_PLA_) and Experimental (HIIT_EXP_) groups.

**Parameters**		**Baseline**	**Post-HIIT**	**Change from baseline to Post-HIIT (%)**	**Cohen’s *d***
Body weight (kg)	HIIT_PLA_	68.8 ± 9.2	68.5 ± 9.1	–0.5	0.03
	HIIT_EXP_	71.0 ± 13.6	69.8 ± 13.6^#⁣‡^	–1.8	0.09
Lean mass (kg)	HIIT_PLA_	48.8 ± 2.9	48.9 ± 3.5	0.2	0.03
	HIIT_EXP_	47.8 ± 7.1	47.8 ± 6.6	0.0	0.00
Body fat (%)	HIIT_PLA_	28.9 ± 3.5	28.9 ± 3.4	–0.2	0.01
	HIIT_EXP_	32.7 ± 3.2	31.2 ± 3.0^#⁣‡^	–4.5	0.48
Hip circumference (cm)	HIIT_PLA_	104.3 ± 3.5	103.6 ± 3.4	–0.7	0.21
	HIIT_EXP_	109.2 ± 9.9	104.7 ± 9.4^#⁣‡^	–4.1	0.47
Waist circumference (cm)	HIIT_PLA_	88.3 ± 7.3	87.6 ± 7.3	–0.8	0.10
	HIIT_EXP_	95.8 ± 9.6	89.3 ± 8.9^#⁣‡^	–6.7	0.70
Body mass index (kg m^–2^)	HIIT_PLA_	25.1 ± 2.9	25.1 ± 2.8	–0.0	0.00
	HIIT_EXP_	26.5 ± 3.3	26.0 ± 3.3†	–1.9	0.15

*^#^Interaction effect (P < 0.01), ^†^difference between Baseline and Post-HIIT (P < 0.05), ^‡^difference between Baseline and Post-HIIT (P < 0.01). Percentage change from Baseline to Post-HIIT. The criteria to interpret the magnitude of the effect size were as follows; >0.2 small, >0.5 moderate, >0.8 large, and >1.3 very large).*

#### Blood Analyses

Changes in biochemical variables are described in Table 4. There was a significant interaction (group × time) effect for TG (*P* < 0.05), with a significant reduction over time for the HIIT_EXP_ group only (−29.2% vs. −6.7% for HIIT_EXP_ and HIIT_PLA__,_ respectively). A significant interaction (group × time) effect was calculated for NEFA (*P* < 0.05) with a decrease Post-HIIT for the HIIT_EXP_ group (−33.9% vs. −5.7% for HIIT_EXP_ and HIIT_PLA__,_ respectively). A significant interaction effect (group × time) for glycerol was recorded (*P* < 0.05) with a decrease in glycerol concentration for HIIT_EXP_ group (−31.1% vs. −8.4% for HIIT_EXP_ and HIIT_PLA__,_ respectively). A significant interaction was recorded for fasting glucose (*P* < 0.05) with a decrease over time for HIIT_EXP_ only (−5.9% vs. 0.6% for HIIT_EXP_ and HIIT_PLA__,_ respectively).

**TABLE 4:** Mean ± SD values for biochemical assessment at Baseline and following the training intervention (Post-HIIT) in Placebo (HIIT_PLA_) and Experimental (HIIT_EXP_) groups.

**Parameters**		**Baseline**	**Post-HIIT**	**Change from baseline to Post-HIIT (%)**	**Cohen’s *d***
Triglycerides (mmol L^–1^)	HIIT_PLA_	1.04 ± 0.27	0.97 ± 0.29	–6.7	0.25
	HIIT_EXP_	1.13 ± 0.48	0.80 ± 0.22^*‡^	–29.2	0.88
Non-esterified fatty acids (μmol L^–1^)	HIIT_PLA_	494 ± 78.1	467 ± 69.2	–5.7	0.16
	HIIT_EXP_	565 ± 101.1	374 ± 87.3^*‡^	–33.9	1.18
Glycerol (mmol L^–1^)	HIIT_PLA_	0.045 ± 0.015	0.041 ± 0.014	–8.4	0.27
	HIIT_EXP_	0.052 ± 0.023	0.036 ± 0.021^*‡^	–31.1	0.73
Fasting glucose (g L^–1^)	HIIT_PLA_	4.95 ± 0.48	4.92 ± 0.36	–0.6	0.07
	HIIT_EXP_	5.11 ± 0.42	4.81 ± 0.39^*†^	–5.9	0.74

*^∗^Interaction effect (*P* < 0.05), ^†^difference between Baseline and Post-HIIT (*P* < 0.05), ^‡^difference between Baseline and Post-HIIT (*P* < 0.01). Percentage change from Baseline to Post-HIIT. The criteria to interpret the magnitude of the effect size were as follows; >0.2 small, >0.5 moderate, >0.8 large, and >1.3 very large).*

### High-Intensity Interval Training (HIIT)

The total weekly training duration was ∼135-min and adherence to training was greater than 95%. The time taken to reach the target heart rate ranged from 20 to 90-s depending on the number of previous efforts completed (Sessions 1 and 2 HIIT_EXP_: 72 ± 28-s; HIIT_PLA_: 78 ± 37-s; Sessions 3 and 4 HIIT_EXP_: 47 ± 19-s; HIIT_PLA_: 43 ± 24-s; Sessions 5 and 6 HIIT_EXP_: 31 ± 17-s; HIIT_PLA_: 28 ± 19-s). Finally, HIIT_EXP_ and HIIT_PLA_ spent about 68.7 ± 10.1% and 69.1 ± 7.9%, respectively, above 90% HRmax for the total training period.

### Change in Subject Satisfaction

The completed CSQ-8 was returned by 18 women (9 in each group, HIIT_EXP_ and HIIT_PLA_), thus displaying a response rate of 90%. The median CSQ-8 after the HIIT program was 29.2 and 16.1 for HIIT_PLA_ and HIIT_EXP_, respectively. Thus, indicating a very high level of satisfaction among women exercising using the diffuse ultrasound compared with the placebo group of women. 92% of HIIT_EXP_ group would have the procedure performed again compared to only 57% for HIIT_PLA_ group. Moreover, the majority (85%) of women in the HIIT_EXP_ group would recommend the program to a friend, but this was only the case for 50% in the HIIT_PLA_ group. In addition, we observed a significant correlation between the level of customer satisfaction in HIIT_EXP_ group and the reduction of circumference measurements obtained after the HIIT program (*r* = −0.88; *P* < 0.05).

## Discussion

The main finding of this study was that HIIT combined with LOFU resulted in a significant reduction in Fat% (−4.5%). Moreover, this improved body composition occurred with significant decreases in plasma TG, NEFA and glycerol in the HIIT_EXP_ group compared to the placebo control group. Both groups improved their fitness level after the HIIT program, as demonstrated by increases in $\dot{\text{V}}$O_2__max_, MAP and F_max_. However, only the experimental group, who trained with LOFU switched on, had positive changes in body composition, girths and lipid profile.

There is a growing body of evidence to support the efficacy of HIIT to improve fitness (Batacan et al., 2017). In the current investigation, six HIIT-sessions over a 2-week period led to significant increases in $\dot{\text{V}}$O_2_max for the previously untrained women in both HIIT_PLA_ and HIIT_EXP_ groups. This finding supports the beneficial effects of short bouts of HIIT on aerobic fitness (Boutcher, 2011) compared to traditional prolonged steady-state training (Nybo et al., 2010). Indeed, when 36 untrained men completed a 12-week program consisting of either intense-interval running (HIIT), strength-training or prolonged steady-state moderate-intensity running (MICT), the HIIT group reported a 14% increase in $\dot{\text{V}}$O_2_max (Nybo et al., 2010). While only a 7% improvement in $\dot{\text{V}}$O_2_max was observed in the MICT group and $\dot{\text{V}}$O_2_max in the strength training group remained unchanged. It is also noteworthy that HIIT elicited this greater increase in fitness level despite participants completing just a third of the total training duration compared to the MICT and strength-training groups. This finding supports the notion that training intensity is more important than training volume for the development of cardiorespiratory fitness (Wenger and Bell, 1986). Several other studies have reported a similar increase in $\dot{\text{V}}$O_2_max in untrained participants to those observed in the present study. For example, Talanian et al. (2007), used a similar protocol of 2 weeks of HIIT and reported a 13% increase in $\dot{\text{V}}$O_2_max in previously untrained women. These improvements in fitness level suggest that a combination of high training intensities (90% HRmax), short durations of each bout (4-min) with intermittent recovery periods provide a strong stimulus for physiological adaptation.

While reductions in fat mass with HIIT have previously been associated with improvements in the fat oxidation pathway (Astorino et al., 2017), in the present investigation, there was only a trend toward a decreased RER across a range of submaximal workloads. This trend was greater in the HIIT_EXP_ compared to the HIIT_PLA_. Prior investigations have linked an improved capacity for fatty acid oxidation following HIIT to an upregulation of key metabolic enzymes within the mitochondria and skeletal muscle (Talanian et al., 2007; Burgomaster et al., 2008; Perry et al., 2008; Boutcher, 2011). This includes a significantly greater expression of the enzyme 3-hydroxyacyl-coenyme A dehydrogenase (Tremblay et al., 1994), citrate synthase (Tremblay et al., 1994; Talanian et al., 2007; Perry et al., 2008), muscle β-hydroxyacyl coenzyme A dehydrogenase (Talanian et al., 2007, 2010; Perry et al., 2008), and total muscle fatty-acid-binding protein (Talanian et al., 2007, 2010). Using a comparable number of HIIT sessions (Katzmarzyk et al., 2001), training volume (10 × 4-min bouts) and intensity (90% HRmax) to the current study, Talanian et al. (2007) reported a significantly improved fat oxidation. However, in this study, and others that have found positive changes in RER, substrate oxidation during exercise was determined using a different protocol (Burgomaster et al., 2008; Perry et al., 2008; Talanian et al., 2010). Rather than measuring RER during graded exercise, a low-intensity prolonged, steady-state bout of exercise was used. This is a limitation of the current investigation as it is possible that changes may have been observed if the participants’ RER was assessed over a longer exercise duration. Conversely, this result may also be explained by the time spent at 90% of HRmax being insufficient to induce whole-body fat oxidation, compared to other short-term (2 to 6 weeks) studies (Burgomaster et al., 2008; Perry et al., 2008).

Similar to the findings in the present study for the HIIT_EXP_ group, HIIT has previously been shown to induce fat loss. For example, Tremblay et al. (1994) noted a reduction in fat mass after 20 weeks of HIIT. Trapp et al. (2008) confirmed these results reporting a 2.5 kg reduction in fat mass following a 15-week HIIT program. However, the same positive changes in body fat mass were not observed in the HIIT_PLA_ group. Despite completing the same HIIT program, fat mass (−1.5%) and body weight were unchanged. Moreover, not all HIIT programs have induced changes in body mass. A meta-analysis identified that collectively, short-term (<12 weeks) HIIT showed no effect on body weight and fat mass in either normal or over-weight populations (Batacan et al., 2017). This suggests that the addition of the LOFU to a HIIT program contributed to the accelerated rate of fat loss observed in the HIIT_EXP_ group.

The novel part of the present study was to add LOFU to a HIIT program. Ultrasound devices have emerged with the increasing demand for non-invasive and safe methods to reduce localized fat (Milanese et al., 2014; Tonucci et al., 2014). In this technique, a dome shaped transducer emits pulsating low frequency, low intensity ultrasound waves that are directed onto a small focal point of unwanted fat tissue. Histological analysis and clinical studies have shown that this focused ultrasonic energy is released specifically in the target subcutaneous adipose tissue without causing damage to blood vessels, nerves, connective tissue, or muscles (Brown et al., 2009). This strong mechanical stimulus creates cavitation (breakdown of fat cell membranes), leading to lipolysis in subcutaneous adipose tissue and a reduction of fat deposits (Milanese et al., 2014; Tonucci et al., 2014). In this context, the reduction in abdominal fat (i.e., localized adipose tissue) after LOFU is mainly the result of mechanical disruption of subcutaneous adipocytes. The release of adipose tissue also leads to a subsequent increase in circulating triglycerides within the interstitial fluid. It has been suggested that once the mechanically disturbed triglycerides are released into the circulation, they follow the normal physiological fat metabolism pathways (Hotta, 2010). Therefore, by wearing an ultrasound device during exercise, these disrupted fat cells may then more easily enter the fat oxidation pathway to be metabolized as a fuel source. This combined with the improved ability to oxidize fat through an augmented hormonal response at both a systemic (i.e., increases in circulating catecholemines and insulin sensitivity) (Boutcher, 2011) and local (i.e., elevated skeletal muscle irisin) (Archundia-Herrera et al., 2017) level associated with HIIT demonstrates how the two modalities can have a synergistic effect on reducing Fat%. Moreover, by having a higher level of circulating free fatty acids this augmented fat oxidation may also continue during the post-exercise recovery period (Greer et al., 2015; Wingfield et al., 2015). The results of the current investigation show strong support for the efficacy of combined HIIT and LOFU to target localized fat.

Significant reductions in circumferences were observed for the HIIT_EXP_ only (waist: −6.5 ± 1.2 cm; hip: −4.6 ± 0.8 cm). These changes in circumference are greater than those previously reported using ultrasound in isolation (Moreno-Moraga et al., 2007; Teitelbaum et al., 2007; Tonucci et al., 2014). For example, Tonucci et al. (2014) observed significant, but smaller reductions of ∼1.5 cm in the waist circumference, ∼2.1 cm in abdominal circumference and ∼1.9 cm in the umbilical circumference following five sessions (60 days) of ultrasound therapy in 20 healthy, sedentary females. Moreno-Moraga et al. (2007) observed a slightly larger 3.95 ± 1.99 cm reduction in circumference following three sessions (monthly visits) in 30 healthy patients. Although, it should be noted that these studies used a lower number of LOFU sessions than utilized in the present investigation. Generally, a reduction in waist circumference is associated with abdominal obesity and is correlated with both visceral and subcutaneous fat (Pou et al., 2009). This is in line with the current findings, where both a reduction in waist circumference and Fat% was observed. Other studies have also shown similar reductions in fat mass with LOFU treatment (Milanese et al., 2014; Shek et al., 2016). For instance, Milanese et al. (2014) completed DXA scans on 28 non-obese women prior to and following a 10-week (2 sessions per week) ultrasound program and found that participants lost an average of −3.4% fat mass, with a reduction of −3.9% in the trunk. Comparable results have been observed with HIIT alone, with a significant reductions in total abdominal and visceral fat mass (Maillard et al., 2018). Nonetheless, considering the sizeable improvements in body composition observed in the current investigation, it appears that there is a synergistic effect of HIIT and LOFU.

A limiting factor of fat oxidation is the release of triglycerides from the adipose tissue (Purdom et al., 2018). With the mechanical stress induced by the LOFU belt, this release is artificially stimulated and is no longer regulated by hormonal or enzymatic factors. HIIT has also been shown to promote increased levels of circulating free fatty acids during and following exercise (Wingfield et al., 2015). It is thought that these increases can promote increase fat oxidation during both exercise and the post-exercise recovery period, leading to body fat loss and an improved lipid profile (Greer et al., 2015). Indeed, in the present investigation there was a tendency toward increased fat oxidation and significant reductions in TG, NEFA, and glycerol concentrations in the HIIT_EXP_ group. While some studies have reported decreases in TG (Elmer et al., 2016) and NEFA (Salvadori et al., 2014), these results have not been consistently observed with HIIT (Batacan et al., 2017). There is also limited evidence in the literature on the impact of LOFU on changes in lipid blood profile as most investigations focus on measures of body composition. Nonetheless, the improvement in lipid profile found in the present study following 2 weeks of exercise is comparable to previous HIIT interventions lasting 8 to 12 weeks (Wisloff et al., 2007). This similar improvement suggests that using LOFU during HIIT can promote greater positive changes in lipid blood markers than exercise alone.

A limitation of this study is that while diet was monitored, it was not strictly controlled for the duration of the training intervention. Both total caloric and macronutrient intake can influence fuel utilization during exercise (Fletcher et al., 2017). Moreover, hunger and hormonal responses were not recorded throughout the training period. However, due to the similar total energy intake reported in both groups, we hypothesized that there was no effect of training or LOFU on appetite or hunger perceptions. Although, an increase in %CHO intake at the end of the HIIT program was observed for the control group, with no change in body fat, body weight, or total calorie intake. In this context, an assessment of eating behaviors may have provided additional insight into their lack of weight loss. As many aspects of eating behaviors are associated with long-term weight control and changes in brain areas implicated in appetite control (McFadden et al., 2013). A deeper understanding of hunger and hormonal responses could have also provided additional insight into the reasons underlying the significant decrease in fat intake (−6.4%) in the HIIT_EXP_ in the second week of training. Another limiting factor, was the lack of non-exercise control group to assess the effects of LOFU in isolation. It would also have been of interest to conduct follow up testing, 1–2 months post-intervention to determine whether the changes in body composition and lipid profile were stable. An additional limitation of the investigation was the use of bioelectrical impedence (BIA) to assess body composition rather than dual-energy x-ray absorptiometry (DXA). The BIA device used in the current investigation has been found to be highly correlated with DXA for measurement of fat mass and Fat% (Verney et al., 2016; Thivel et al., 2018). However, less agreement has been reported between the BIA and DXA in the measurement of muscle mass and a lower reliability has been observed with a high initial body weight (Thivel et al., 2018). As changes in fat mass were the key parameter and the groups were closely matched when the present study commenced, BIA was considered a viable and cost-effective alternative to using DXA to assess body composition. Nonetheless, taken together the collective findings of the study indicate that the combination of HIIT and LOFU can positively impact health, fitness, body composition and that further research into the exercise intervention is warranted.

The high level of satisfaction among women exercising with LOFU further supports future investigation into the implementation of HIIT and LOFU. In the HIIT_EXP_ group 91.7% would complete the program again compared to only 57.1% for HIIT_PLA_ group. These levels of satisfaction are higher than those reported using LOFU alone (Tonucci et al., 2014). This may be due to the observable changes in body composition with combined HIIT and LOFU in a shorter time period. Considering, previous LOFU studies have provided less LOFU treatments over a longer time period (Moreno-Moraga et al., 2007; Tonucci et al., 2014). Moreover, the reduction in circumference measurements was strongly correlated with participant satisfaction (*r* = −0.88) in the HIIT_EXP_ group. These findings suggest that the level of satisfaction is high when participants experienced significant improvements in body composition following a short-term intervention.

In summary, a 2-week, six session HIIT program combined with LOFU was an efficient strategy to increase fitness level and improve body composition in sedentary overweight women. Based on the findings of the present study, it appears that the addition of LOFU to HIIT can further increase the mobilization of free fatty acids from adipose tissue leading to greater losses in fat mass. This combined approach also accelerated positive health outcomes, such as an improved lipid profile and reductions in waist and hip circumference in a short-term intervention. Further research is required to confirm these novel findings and optimize the use of this strategy in both men and women.

## Data Availability Statement

The datasets generated for this study are available on request to the corresponding author.

## Ethics Statement

The studies involving human participants were reviewed and approved by the University of Nice. The patients/participants provided their written informed consent to participate in this study.

## Author Contributions

CH designed the research and project management. L-AM and XN collected and analyzed the data. KS prepared and edited the manuscript.

## Conflict of Interest

XN is employed by the BESCORED company, which has no link with other companies. The remaining authors declare that the research was conducted in the absence of any commercial or financial relationships that could be construed as a potential conflict of interest.
